# Antiviral Property of the Fungal Metabolite 3-*O*-Methylfunicone in Bovine Herpesvirus 1 Infection

**DOI:** 10.3390/microorganisms10010188

**Published:** 2022-01-15

**Authors:** Filomena Fiorito, Claudia Cerracchio, Maria Michela Salvatore, Francesco Serra, Alessia Pucciarelli, Maria Grazia Amoroso, Rosario Nicoletti, Anna Andolfi

**Affiliations:** 1 Department of Veterinary Medicine and Animal Production, University of Naples Federico II, 80137 Naples, Naples, Italy; claudiacerrakkio@gmail.com (C.C.); alessia20-07@hotmail.it (A.P.); 2 BAT Center-Interuniversity Center for Studies on Bioinspired Agro-Environmental Technology, University of Naples Federico II, 80055 Portici, Naples, Italy; 3 Department of Chemical Sciences, University of Naples Federico II, 80126 Naples, Naples, Italy; mariamichela.salvatore@unina.it; 4 Institute for Sustainable Plant Protection, National Research Council, 80055 Portici, Naples, Italy; 5 Istituto Zooprofilattico del Mezzogiorno, 80055 Portici, Naples, Italy; francesco.serra@izsmportici.it; 6 Council for Agricultural Research and Economics, Research Centre for Olive, Fruit and Citrus Crops, 81100 Caserta, Caserta, Italy; rosario.nicoletti@crea.gov.it; 7 Department of Agricultural Sciences, University of Naples Federico II, 80055 Portici, Naples, Italy

**Keywords:** BoHV-1, 3-*O*-methylfunicone, *Talaromyces pinophilus*, MDBK, bICP0, AhR

## Abstract

Bovine herpesvirus type-1 (BoHV-1) is a widespread pathogen that provokes infectious rhinotracheitis and polymicrobial infections in cattle, resulting in serious economic losses to the farm animal industry and trade restrictions. To date, non-toxic active drugs against BoHV-1 are not available. The exploitation of bioactive properties of microbial products is of great pharmaceutical interest. In fact, fungi are a promising source of novel drugs with a broad spectrum of activities and functions, including antiviral properties. Hence, the potential antiviral properties of 3-*O*-methylfunicone (OMF), a secondary metabolite produced by *Talaromyces pinophilus*, were evaluated on BoHV-1. In this study, during BoHV-1 infection in bovine cells (MDBK), the non-toxic concentration of 5 µM OMF considerably reduced signs of cell death and increased cell proliferation. Furthermore, OMF significantly decreased the virus titer as well as the cytopathic effect and strongly inhibited the expression of bICP0, the major regulatory protein in the BoHV-1 lytic cycle. These findings were accompanied by a considerable up-regulation in the expression of the aryl hydrocarbon receptor (AhR), a multifunctional transcription factor also linked to the host’s response to a herpesvirus infection. Overall, our results suggest that by involving AhR, OMF shows potential against a BoHV-1 infection.

## 1. Introduction

Bovine herpesvirus 1 (BoHV-1), a pathogen belonging to the alphaherpesvirus subfamily, is responsible for considerable economic losses to the cattle industry as well as trade restrictions [1,2]. Because of its immunosuppressive features, it may cause infectious bovine rhinotracheitis, vulvovaginitis, abortions, or polymicrobial infections. Indeed, BoHV-1 is an important cofactor for the bovine respiratory disease complex, the most important inflammatory disease in cattle, causing pneumonia and sometimes death [1,2]. BoHV-1 establishes latency in sensory neurons of the infected host, and reactivation from latency is induced by corticosteroids, developing virus shedding, and spread to susceptible hosts [1,2]. Similar to other members of the alphaherpesvirus subfamily, gene expression of BoHV-1 occurs in three temporally different phases known as immediate-early, early, and late; tissue-specific elements are involved in pathogenesis and/or in latency by affecting viral gene expression [1,2]. bICP0, the bovine homologue of the herpes simplex virus type 1 (HSV-1) ICP0, controls these three phases through a strong activation or repression of certain viral promoters. bICP0 is the major regulatory protein that stimulates productive infection; it is expressed during infection in permissive cells and inhibits interferon-dependent transcription [2,3,4,5].

To date, non-toxic active drugs against BoHV-1 are not available, but plant and fungal extracts are of great interest for the development of new antiviral drugs [6,7,8,9,10,11]. More specifically, some of these products, such as peptides derived from a strain of *Scytalidium* sp. [12], macrolides derived from an unidentified fungus belonging to the Pleosporales [13], and lactones derived from a strain of *Aspergillus terreus* [14], have shown inhibitory activity against herpesviruses.

In relation to their great adaptability to the most varied habitats and lifestyles, fungi in the genus *Talaromyces* (Eurotiales: Trichocomaceae) are characterized by high biosynthetic versatility and have been reported as a source of many bioactive products of pharmaceutical interest, including antiviral drugs [15,16,17,18].

**Figure 1:**
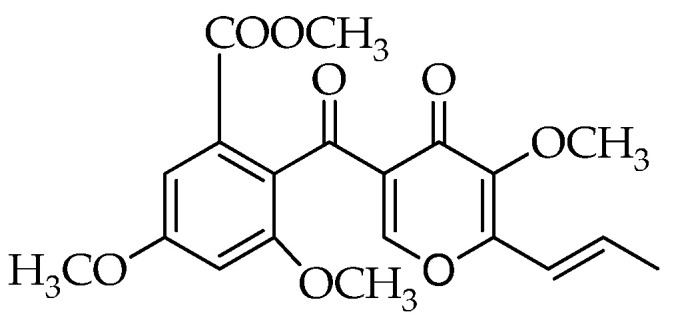
Structure of 3-*O*-methylfunicone (OMF).

The emphasis in current literature is, in particular, on *Talaromyces pinophilus*, previously known as *Penicillium pinophilum*. In fact, its antagonistic behavior has been reported in relation to the production of 3-*O*-methylfunicone (OMF), a benzo-*γ*-pyrone compound first characterized for its in vitro inhibitory effects on some plant pathogenic fungi (Figure 1) [19,20]. Subsequently, several studies reported the antiproliferative and proapoptotic proprieties of this compound on tumor cells [21,22,23], which shed light on its potential use as a cancer therapeutic. In a screening conducted on some analogues of the funicone series, it was observed that OMF reduced infectivity of hepatitis C virus (HCV) [24]. Despite the relevance of its biological properties, pathways underlying the biosynthesis of OMF have not been explored in depth. However, the recent genome sequencing of *T. pinophilus* has paved the way for the elucidation of the biochemical processes leading to the production of this secondary metabolite [25].

The aryl hydrocarbon receptor (AhR) is a ligand-activated transcription factor. It acts together with many endogenous and exogenous substances, involving bilirubin, biliverdin, tryptophan metabolites, environmental pollutants (dioxin), and microbial metabolites [26]. After the activation, AhR translocases into the nucleus, where it controls the expression of target genes, such as the AhR repressor, detoxifying monooxygenases (CYP1A1 and CYP1B1), and cytokines. AhR is also involved in cellular mechanisms, such as proliferation and apoptosis, immune modulation, and other processes, which further affect cell growth, survival, migration, and invasion. In addition, recent evidence indicates that some agonists of AhR might improve the host response to a herpesvirus infection [27].

Until now, no circumstantial study has been performed to assess the antiviral properties of OMF, and BoHV-1 represents a good model for anti-herpesvirus molecule screening, as previously described [28,29,30]. Based on these premises, this study aimed to evaluate the antiviral activity of OMF on BoHV-1.

## 2. Materials and Methods

### 2.1. Production of OMF

The OMF used in this study was extracted from an isolated LT6 of *T. pinophilus*, previously recovered from the rhizosphere of tobacco (*Nicotiana tabacum*), producing this funicone compound along with a new macrodiolide, named talarodiolide, and several known metabolites [31]. The liquid fungal culture was prepared by inoculating mycelial plugs from actively growing cultures in 1 L Erlenmeyer flasks containing 500 mL of potato dextrose broth (PDB, Himedia), which were kept in darkness on a stationary phase at 25 °C. After 21 days, cultures were filtered at 0.45 µm and extracted with ethyl acetate. The crude extract was submitted to fractionation by column chromatography and thin-layer chromatography on silica gel, eluting with diverse polarity solvents. OMF was identified by comparing the NMR data with previous reports [31].

### 2.2. Cell Cultures and Virus Infection

Madin Darby Bovine Kidney (MDBK), a bovine cell line (American Type Culture Collection, CCL22), was cultured in Dulbecco’s modified Eagle’s minimal essential medium (DMEM) and incubated at 37 °C and 5% CO_2_, as previously described [5]. BoHV-1 (Cooper strain) was utilized throughout the study. MDBK cells were used for virus stock growth and virus titration [32,33,34].

OMF was dissolved in dimethyl sulfoxide (DMSO) and added to the medium at a final concentration of 1, 5, 10, 25, and 50 μM. Monolayers of MDBK cells were either infected or not infected with BoHV-1, at a multiplicity of infection (MOI) of 0.1, 1, or 5, in the presence or absence of OMF, to obtain four groups: untreated uninfected cells; untreated infected cells; OMF-treated uninfected cells; OMF-treated infected cells. After 1 h of adsorption at 37 °C, cells were incubated and processed at 1, 3, 6, 12, and 24 h post-infection (p.i.). The virus was in a culture medium through the course of the experiment.

### 2.3. Cell Viability

To assess cell viability, the trypan blue (TB) (Sigma-Aldrich, Milan, Italy) exclusion test was used, as previously described [35]. Briefly, monolayers of MDBK cells were either infected or not infected with BoHV-1, at an MOI of 5, in the presence or absence of OMF at different concentrations (1, 5, 10, 25, and 50 μM) to obtain four groups: untreated uninfected cells; untreated infected cells; OMF-treated uninfected cells; OMF-treated infected cells. After 24 h of treatment, cells were collected by trypsinization, mixed with TB, and scored through the TC20 automated cell counter (Bio-Rad Laboratories S.r.l., Segrate, Milan, Italy). Cell viability was calculated as the percentage of living cells over the total cell number. The results are reported as the mean ± S.D. of three independent experiments performed twice. Furthermore, cell viability was evaluated by the TB in cells attached to wells, as described [33,36].

### 2.4. Cell Proliferation

To evaluate cell proliferation, a 3-(4,5-dimethyl-2-thiazolyl)-2,5-diphenyl-2H-tetrazolium bromide (MTT) assay was used, as previously described [5,35,37]. Briefly, MDBK cultured in 96-well plates were infected with BoHV-1, at an MOI of 1, exposed or not exposed to OMF (5 µM), and incubated. At 24 h p.i., an MTT assay was carried out. The results were the mean ± S.D. of four independent experiments performed twice.

### 2.5. Examination of Cell Morphology

To examine cell morphology, light microscopy following Giemsa staining was used [33]. Briefly, the monolayers of MDBK cells were either infected or not infected with BoHV-1, at an MOI of 1, in the presence or absence of OMF (5 µM), and incubated at 37 °C. After 24 h p.i., Giemsa staining was performed, and a light microscopy examination was carried out under the ZOE Cell Imager (Bio-Rad Laboratories). The cell death features were identified by using the criteria previously described [38,39,40].

### 2.6. Immunofluorescence Staining

MDBK cells were either infected or not infected with BoHV-1, at an MOI of 0.1, and treated with OMF (5 µM). After 24 h p.i., immunofluorescence staining was performed as previously reported [41], by using the following antibodies dissolved in 5% bovine serum albumin-TBST: anti-AhR (Sigma-Aldrich) (1:250); anti-bICP0 polyclonal rabbit (a.a. 663–676) serum (1:800), kindly provided by Prof. M. Schwyzer and Prof. Cornel Fraefel (University of Zurich, Zurich, Switzerland); and Texas Red goat anti-rabbit (Thermo Fisher Scientific, Waltham, MA, USA) (1:100). Nuclear counter-staining was evaluated by DAPI (1:1000). Microscopy and photography were assessed by the ZOE Fluorescent Cell Imager (Bio-Rad Laboratories, Hercules, CA, USA). Quantification of fluorescence signals from microscopy-generated images were performed using ImageJ (National Institutes of Health, Bethesda, MD, USA) software.

### 2.7. Virus Production

MDBK cells were infected with BoHV-1, at an MOI of 1, in the presence or absence of OMF, incubated at 37 °C, and processed at 0, 1, 3, 6, 12, and 24 h p.i. by real-time PCR for BoHV-1 quantification. Furthermore, the virus titer was evaluated in MDBK cells by the TCID_50_ method, as previously described [33]. In addition, viral cytopathic effects (CPE) were evaluated. To this purpose, cells were examined under a light microscope at indicated times of infection, as previously described [34].

### 2.8. Viral Nucleic Acids Extraction Procedures

Nucleic acids extraction was carried out from 200 µL of cell supernatant using the King Fisher Flex System (Thermo Fisher Scientific, Waltham, MA, USA) with the Mag Max Viral Pathogen kit (Thermo Fisher Scientific, Waltham, MA, USA), according to the instructions of the manufacturer. Nucleic acids were dissolved in 80 µL of elution buffer. DMEM was used as negative process control.

### 2.9. Real-Time PCR for Quantification of BoHV-1

BoHV-1 was quantified in all the samples by quantitative real-time PCR. Detection was carried out on a Quant Studio 5 Real-Time PCR thermal cycler (Thermo Fisher Scientific, Waltham, MA, USA) in a total volume of 25 µL containing 5 µL of nucleic acids extract, 12.5 µL of TaqMan Universal PCR Master Mix 2X (Thermo Fisher Scientific, Waltham, MA, USA), 1 µL (4.5 µM) of primer forward gBF (5′- TGTGGACCTAAACCTCACGGT-3′), 1 µL (4.5 µM) of primer reverse gBR (5′-GTAGTCGAGCAGACCCGTGTC-3′), and 1 µL (3 µM) of probe gB-P (FAM-5′-AGGACCGCGAGTTCTTGCCGC-3′-TAMRA). The thermal profile was initial denaturation for 15 min at 95 °C and 45 cycles of amplification for 15 s at 95 °C and for 45 s at 60 °C (OIE Manual of Terrestrial Animals Cap. 3.4.11. par B.1.3.1 2017).

Quantification was carried out by a standard curve, analyzing serial dilutions of the quantified extracted virus (from 1 × 10^7^ to 1 × 10 TCID_50_/mL) and plotting the TCID_50_/mL versus the threshold cycle (Ct).

### 2.10. Statistical Analysis

Data are presented as mean ± S.D. One-way ANOVA with Tukey’s post-test was performed by GraphPad InStat Version 3.00 for Windows 95 (GraphPad Software, San Diego, CA, USA). *p* < 0.05 was considered statistically significant.

## 3. Results

### 3.1. OMF Decreases Cell Death during BoHV-1 Infection

To investigate the biological influence of OMF during BoHV-1 infection in MDBK cells, we first evaluated cell viability by TB exclusion test, and then we tested cell proliferation by MTT. As described above, identifying IC_50_ of OMF and developing a dose–response curve was carried out by treating MDBK cells with different doses of OMF. Thus, monolayers of MDBK cells were either infected or not infected with BoHV-1, at an MOI of 5, in the presence or absence of OMF at different concentrations (1, 5, 10, 25, and 50 μM) to obtain four groups: untreated uninfected cells; untreated infected cells; OMF-treated uninfected cells; OMF-treated infected cells. 

**Figure 2:**
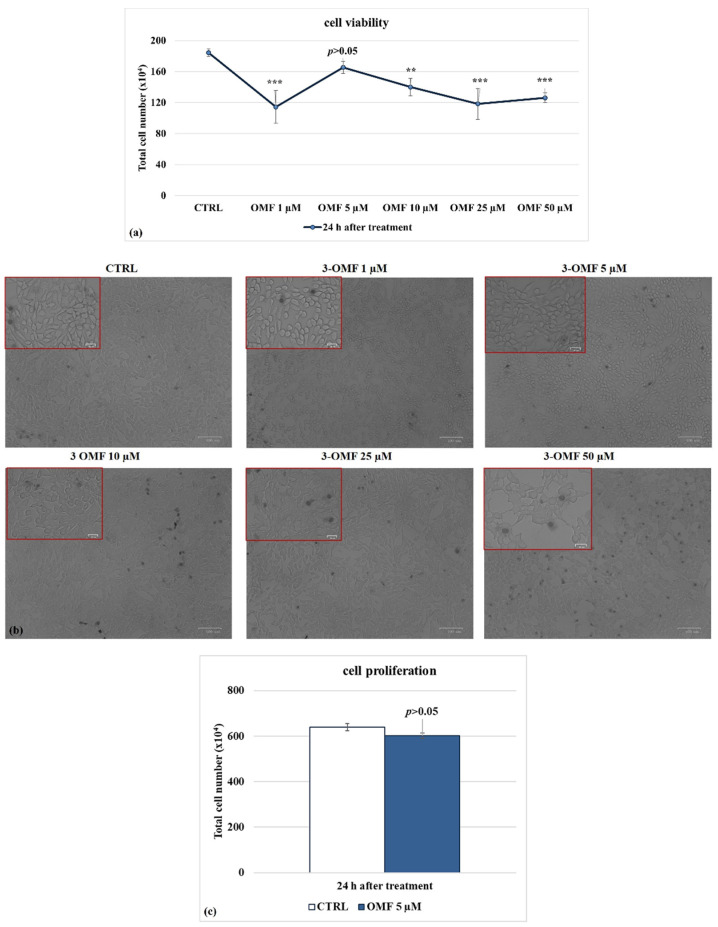
Identifying IC_50_ of OMF at different doses and developing dose–response curve in MDBK cells. (**a**) Dose–response curve of MDBK cells treated with OMF at different concentrations (1, 5, 10, 25, and 50 μM). At 24 h after treatment, cells were stained with TB and scored by automated cell counter, or (**b**) cell viability was determined by TB staining while cells were attached to wells and counted under a light microscope. (**c**) Dose–response curve of MDBK cells treated with OMF (5 μM) for 24 h and assessed by MTT assay. Significant differences between control and OMF-treated cells are indicated by probability *p*. ** *p* < 0.01 and *** *p* < 0.001. Scale bar 100 µm.

Dose-dependent inhibition of cell growth was detected in MDBK cells with an IC_50_ of about 10 μM OMF at 24 h (Figure 2a). OMF at 5 µM produced no significant differences in cell viability (*p* > 0.05) in MDBK cells (Figure 2a). Similar results were found on cell viability, analyzed using TB staining while cells were attached to wells and scored under a light microscope (Figure 2b).

To explore the effect of OMF on MDBK cells’ proliferation, we analyzed the mitochondrial redox ability by MTT assay. At 24 h p.i., OMF at 5 μM did not provoke significant (*p* > 0.05) time-dependent alteration in the amount of mitochondrial dehydrogenases activity compared to control cells (Figure 2c). In brief, these experiments showed that OMF at the concentration of 5 μM did not significantly modify MDBK cell viability and cell proliferation as compared to untreated cells.

**Figure 3:**
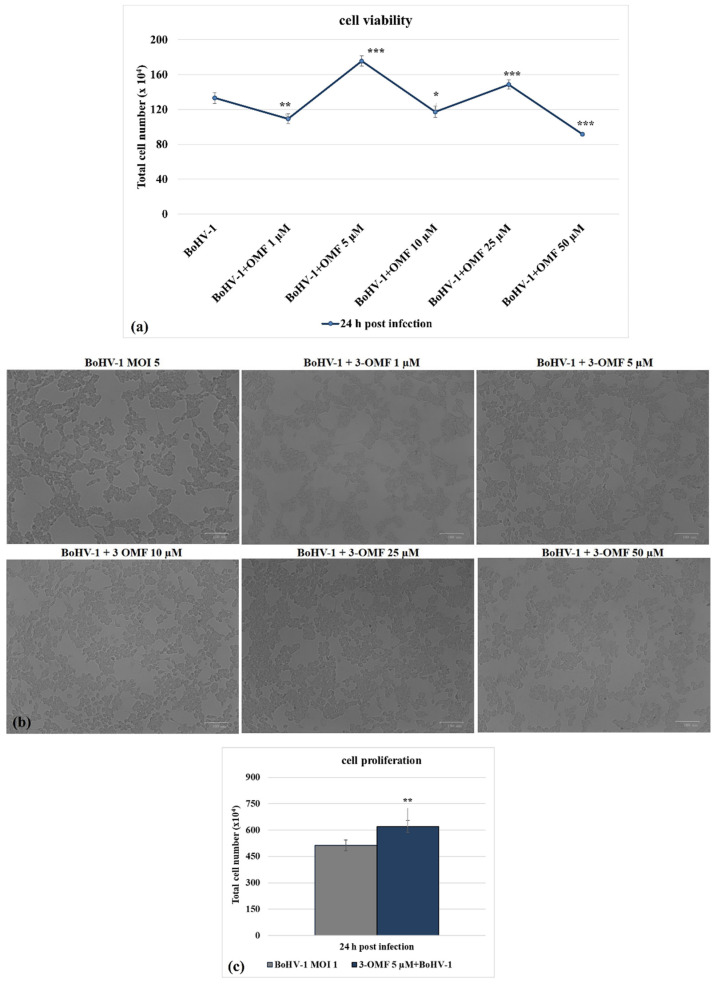
OMF decreases cell death during BoHV-1 infection. (**a**) Dose–response curve of MDBK cells infected with BoHV-1 and treated with OMF at different concentrations (1, 5, 10, 25, and 50 μM). At 24 h after treatment, cells were stained with TB and scored by an automated counter, or (**b**) cell viability was assayed by using TB staining while cells were attached to wells and counted under a light microscope. (**c**) Dose–response curve of MDBK cells infected with BoHV-1, treated with OMF (5 μM) for 24 h and analyzed by MTT assay. Significant differences between control and OMF-treated cells are indicated by probability *p*. * *p* < 0.05, ** *p* < 0.01, and *** *p* < 0.001. Scale bar 100 µm.

Following BoHV-1 infection, in the presence of OMF at 5 µM, there was significantly (*p* < 0.001) decreased cytotoxicity (Figure 3a,b) and increased cell proliferation (*p* < 0.01) during infection in MDBK cells (Figure 3c). Thus, we selected the concentration of OMF at 5 µM for use throughout the study. Overall, at the non-toxic concentration of 5 µM, OMF significantly decreased the MDBK cell death at 24 h p.i. during BoHV-1 infection.

### 3.2. OMF Decreases Cell Membrane Damage and Morphological Cell Death Features during BoHV-1 Infection in MDBK Cells

**Figure 4:**
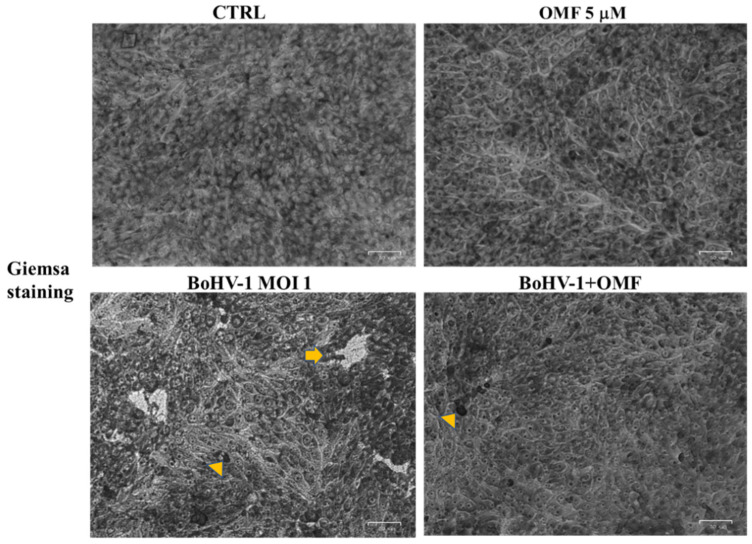
OMF decreases cell membrane damage and morphological cell death features during BoHV-1 infection in MDBK cells. Cells were infected with BoHV-1, in the presence or absence of OMF. At 24 h p.i., cells after Giemsa staining were examined under a light microscope. Photomicrographs showed no morphological alterations in OMF unexposed groups compared to control. By comparing BoHV-1-infected cells to the control, some cells displayed apoptotic features, attributable to pyknotic nuclei and nuclear fragmentation (arrow), or necrosis marks, such as nuclear and cytoplasmic swelling (arrowhead). In OMF-treated infected cells, only a few signs of necrosis were found (arrowhead). Scale bar 50 µm.

To detect cell morphology, the effects of OMF in BoHV-1-infected cells were examined by light microscopy after Giemsa staining. After 24 h of infection, no morphological alterations were found in the OMF unexposed groups compared to the control, as shown in Figure 4. In unexposed infected cells, we observed a growth of intercellular spaces and alteration in morphology, indicating marks of apoptotic cell death due to cell shrinkage, pyknosis, chromatin condensation, and fragmentation of nuclei (Figure 4, arrow). These features were accompanied by necrosis because of nuclear and cytoplasmic swelling due to a break of the plasma membrane, provoking a decreased definition of cell shapes (Figure 4, arrowhead). While only a few signs of necrosis were observed in BoHV-1-infected cells treated with OMF (Figure 4, arrowhead), these results showed that MDBK cells infected with BoHV-1, in the presence of OMF, did not undergo either apoptotic or necrotic cell death.

### 3.3. OMF Decreases Virus Yield and Reduces the Expression of bICP0 during BoHV-1 Infection

Following BoHV-1 infection for 24 h in MDBK cells, we analyzed virus titer and viral CPE to explore the effect of OMF on virus production. Thus, MDBK cells were infected with BoHV-1, at an MOI of 1, in the presence or absence of OMF at the non-toxic concentration of 5 µM and processed.

**Figure 5:**
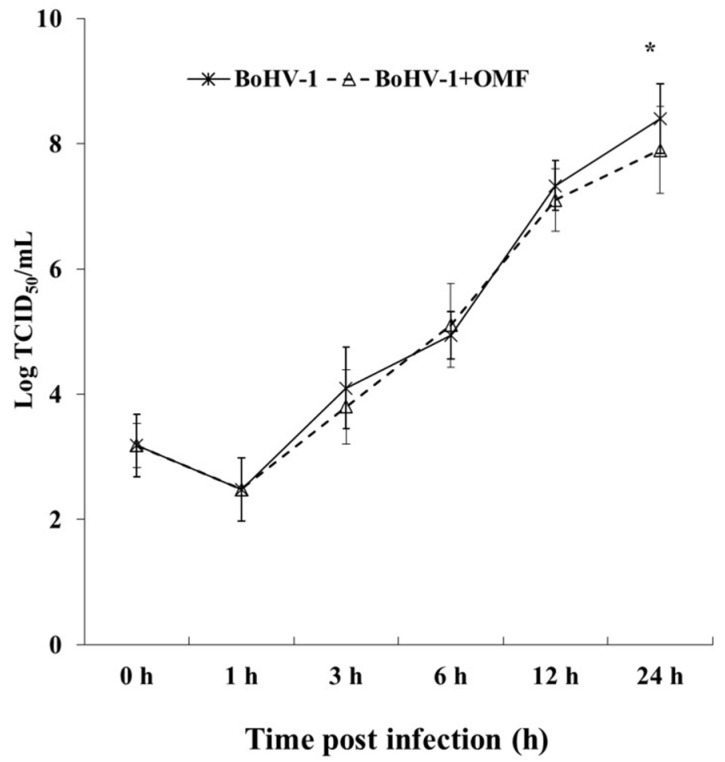
OMF decreases virus yield during BoHV-1 infection in MDBK cells. Cells were infected with BoHV-1 in the presence or absence of OMF. For viral growth curves, MDBK cells were infected with BoHV-1 in the presence or absence of OMF. At indicated times, p.i., virus titer was evaluated by real-time PCR. Significant differences between BoHV-1-infected cells and OMF-treated infected cells are indicated by probability *p*. * *p* < 0.05.

Interestingly, a statistically significant (*p* < 0.05) decrease in the virus titer was seen at 24 h p.i. in cells treated with OMF during BoHV-1 infection (Figure 5). Similar results were found carrying out the virus titer by TCID_50_ (data not shown). Moreover, at 24 h p.i., CPE, due to syncytia development as well as damaged cellular sheet, was considerable in infected groups, while it was markedly decreased in OMF-treated infected cells (Figure 4). Hence, our results showed that OMF considerably reduces virus yield as well as CPE due to BoHV-1 infection.

**Figure 6:**
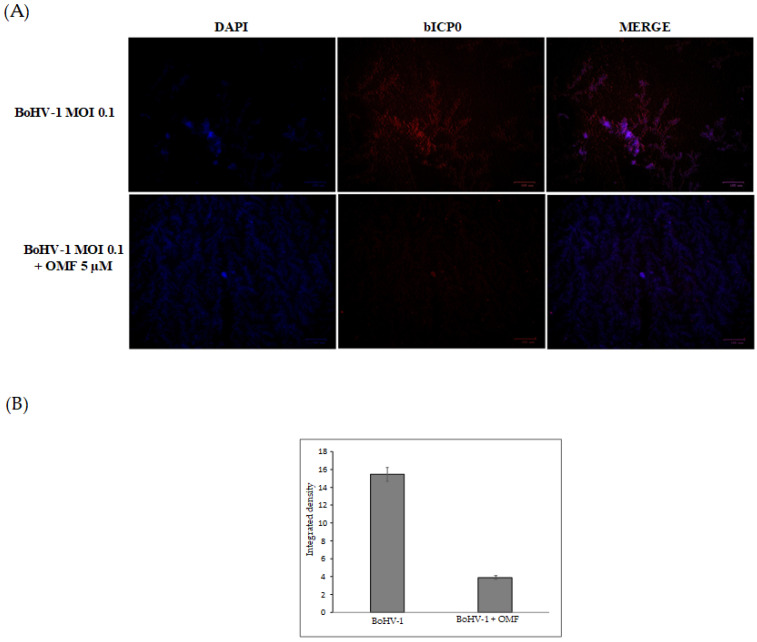
OMF reduces the expression of bICP0 during BoHV-1 infection in MDBK cells. (**A**) Cells were infected with BoHV-1, at an MOI of 0.1, in the presence or absence of OMF, and after 24 h p.i., immunofluorescence staining for bICP0 (red fluorescence) was performed as described in the Methods section. Nuclei were counterstained with DAPI. During infection, bICP0 was localized in both the nucleus and cytoplasm. In the presence of OMF, the expression of bICP0 was noticeably reduced during BoHV-1 infection. Scale bar 100 µm. (**B**) Bars represent the mean ratio generated from the integrated density (product of the area and mean intensity of fluorescence) of the bICP0 expression evaluated by ImageJ. Error bars represent standard error measurement.

To further clarify the effects of OMF during BoHV-1 infection, we analyzed bICP0 protein expression, the main protein involved in the transcription of BoHV-1 [1,2]. Indeed, bICP0 is expressed during infection in permissive cells, in which it promotes productive infection [3,4,42,43,44]. At 24 h p.i., bICP0 was found in BoHV-1-infected cells (Figure 6), whereas the protein was considerably decreased in the presence of OMF (5 µM) (Figure 6). These results demonstrate that the bICP0 viral protein is generally expressed in BoHV-1-infected cells, and its expression was reduced in infected cells treated with OMF.

### 3.4. OMF Induces the Expression of AhR during BoHV-1 Infection in MDBK Cells

**Figure 7:**
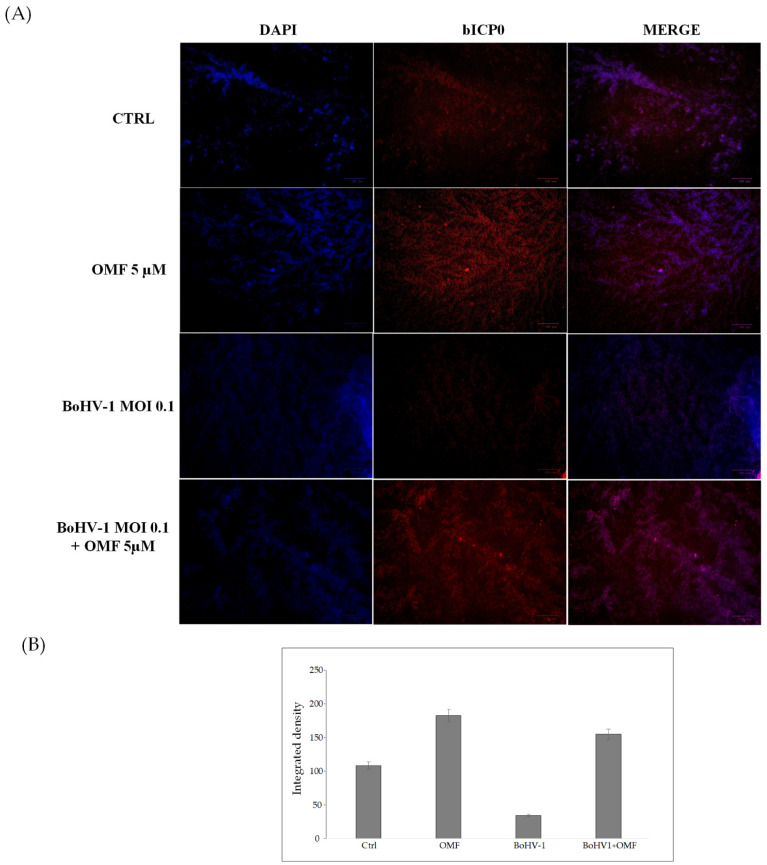
OMF induces the expression of AhR during BoHV-1 infection in MDBK cells. (**A**) Representative microphotographs of uninfected (control) or infected cells (BoHV-1), OMF-treated infected (BoHV-1+OMF), and uninfected cells (OMF), after 24 h of infection, stained with immunofluorescence for AhR (red fluorescence), as described in the Methods section. Nuclei were counterstained with DAPI. AhR was expressed in MDBK cells and localized in both the nucleus and cytoplasm. The expression of AhR was induced in the presence of OMF. During infection, the expression of AhR was drastically reduced. In the presence of OMF, the expression of AhR was remarkably enhanced during BoHV-1 infection. Scale bar 100 µ (**B**) Bars represent the mean ratio generated from the total integrated density (product of the area and mean intensity of fluorescence) of the AhR expression evaluated by ImageJ. Error bars represent standard error measurement.

To explore the potential involvement of OMF in the regulation of AhR, we carried out an immunofluorescence assay for AhR, a receptor expressed in MDBK cells [45]. Herein, OMF induced the activation of AhR in MDBK cells, and, following infection with BoHV-1, a noteworthy activation of AhR in OMF-treated cells was detected (Figure 7).

Our results showed that the AhR cellular receptor, generally expressed in MDBK cells, was activated in the presence of OMF. In addition, the expression of AhR strongly increased in infected cells treated with OMF.

## 4. Discussions

Many fungal metabolites have caught the attention of researchers involved in the development of novel therapeutic strategies against viral diseases. In fact, they might represent an attractive tool to be exploited with reference to their diverse structures and mechanisms of action. The available data concerning bioactivity of fungal products or extracts against herpes viruses are limited, but it is worth noting that some compounds, including butyrolactone derivatives, macrolides, naphthalenones, resorcylic acid lactones, sterols, and peptides, may act as anti-HSV agents [6,7,46]. The structural variability of fungal metabolites with anti-HSV activity might be related to different mechanisms of action, but there is a lack of information on this aspect. It was hypothesized that the lipophilic linear peptides, halovirs, could destabilize the virus membrane [12].

Herein, we explored the effect of OMF during a BoHV-1 infection. In MDBK cells, OMF offered good protection against virus action by inducing a significant increase in cell viability as well as in cell proliferation. Typically, BoHV-1 stimulates cell death in a cell-type-dependent trend [33,47], and the morphological evaluation of MDBK during infection revealed a decrease in cell membrane damage and cell death features, which are considerably reduced by OMF. Similar signs of MDBK cell protection during BoHV-1 infection were previously detected in the presence of the proteasome inhibitor MG-132 [28]. Similarly, a decrease in virus replication in OMF-treated infected cells was found. Indeed, a reduction in CPE and a significant decrease in virus yield were detected. Interestingly, these results were accompanied by a marked reduction in bICP0 expression, indicating that the virus discharge was considerably reduced by OMF.

Concerning the BoHV-1, there are some reports of new potential agents with inhibitory effects against this virus, but they are often classical synthetic agents (acyclovir, famciclovir, fenbendazole, and ivermectin) used alone or in combination with natural products [29,30]. Inference of bioactivity of fungal products against this virus was derived from the evaluation of extracts of the mushrooms *Agaricus blazei* [48] and *Lentinula edodes* [49]. Hence, the OMF inhibitory activity against BoHV-1 observed in the present investigation represents the first evidence concerning a purified metabolite. So far, anti-HCV properties of this compound were reported in a comparative study where the examination of two funicone derivatives suggested that the 1,3-dihydroxy-5-methylbenzene moiety is important for antiviral activity [24]. It is possible that the arylic nature of this part of the molecule interacts with AhR, a ligand-activated transcription factor that interacts with aromatic compounds, but this hypothetical interaction should be further investigated.

AhR has been described as a multifunctional sensor, integrator system, and ligand-activated transcription factor of the bHLH/PAS family. In the complexity of signaling, AhR regulates elements of the innate and adaptive immune response to various microorganisms. Moreover, AhR is involved in the modulation of the host response to viral infection. Furthermore, the well-known activation of AhR by 2,3,7,8-tetrachlorodibenzo-*p*-dioxin (TCDD or dioxin) may induce a generalized suppression of immune response and increase susceptibility to infectious agents, such as viruses, both in vivo and in vitro [44,50]. Specifically, the AhR activation has been shown to promote viral replication of several herpesviruses, such as cytomegalovirus (CMV), herpes simplex II, and BoHV-1. In addition, dioxin may stimulate a worsening of latent infection caused by CMV or Epstein-Barr virus [44]. Similar responses were detected during HSV-1 infection in mice [51]. In fact, dioxin-treated mice had higher virus titers, and many of them died due to herpes encephalitis if AhR was stimulated before to infection. Interestingly, if AhR activation occurred after HSV-1 infection, the pathogenic signs, such as herpes encephalitis, diminished, and eye tissue pathology improved [51], highlighting the importance of timing in AhR stimulation to regulate the balance between reducing immunopathology and removing antiviral defensive immunity [27].

Taken together, our results showed a potential antiviral role of OMF during BoHV-1 infection in MDBK cells by involving AhR. Consequently, AhR targeted therapy may become a new method for antiviral treatment.

## Data Availability

The data that support the findings of this study are available from the corresponding author upon reasonable request.
